# Intraocular Pressure-Lowering Effects of Commonly Used Fixed-Combination Drugs with Timolol: A Systematic Review and Meta-Analysis

**DOI:** 10.1371/journal.pone.0045079

**Published:** 2012-09-13

**Authors:** Jin-Wei Cheng, Shi-Wei Cheng, Lian-Di Gao, Guo-Cai Lu, Rui-Li Wei

**Affiliations:** 1 Department of Ophthalmology, Shanghai Changzheng Hospital, Second Military Medical University, Shanghai, China; 2 School of Life Sciences, Ludong University, Yantai, China; 3 Center for New Drug Evaluation, Institute of Basic Medical Science, Second Military Medical University, Shanghai, China; Duke University, United States of America

## Abstract

**Background:**

The first goal of medical therapy in glaucoma is to reduce intraocular pressure (IOP), and the fixed-combination medications are needed to achieve sufficiently low target IOP. The aim of this systematic review and meta-analysis is to evaluate IOP-lowering effect of the commonly used fixed-combination drugs containing 0.5% timolol.

**Methods:**

Pertinent publications were identified through systematic searches. Over 85% of the patients had to be diagnosed with primary open-angle glaucoma (POAG) or ocular hypertension (OHT). Forty-one randomized clinical trials were included in the meta-analysis. The main efficacy measures were the absolute and relative values of mean diurnal IOP reduction, and the highest and lowest IOP reductions on the diurnal IOP curve. The pooled 1- to 3-month IOP-lowering effects after a medicine-free washout period was calculated by performing meta-analysis using the random effects model, and relative treatment effects among different fixed combinations were assessed using a mixed-effects meta-regression model.

**Results:**

The relative reductions for mean diurnal IOP were 34.9% for travoprost/timolol, 34.3% for bimatoprost/timolol, 33.9% for latanoprost/timolol, 32.7% for brinzolamide/timolol, 29.9% for dorzolamide/timolol, and 28.1% for brimonidine/timolol. For the highest IOP decrease, relative reductions ranged from 31.3% for dorzolamide/timolol to 35.5% for travoprost/timolol; for the lowest IOP decrease, those varied from 25.9% for dorzolamide/timolol to 33.1% for bimatoprost/timolol. Both latanoprost/timolol and travoprost/timolol were more effective in lowering mean diurnal IOP than brimonidine/timolol (WMD: 5.9 and 7.0) and dorzolamide/timolol (WMD: 3.8 and 3.3).

**Conclusions:**

All six commonly used fixed-combination drugs containing timolol can effectively lower IOP in patients with POAG and OHT, and both latanoprost/timolol and travoprost/timolol might achieve better IOP-lowering effects among the six fixed-combination agents.

## Introduction

Glaucoma has been established as the second leading cause of world blindness, which may affect 60.5 million people worldwide in 2010, and 79.6 million in 2020, and approximately 74% of glaucoma patients have primary open-angle glaucoma (POAG) [1]. The treatment of glaucoma focuses mainly on lowering intraocular pressure (IOP) [2]. The target IOP is often set to a level 20% to 30% of IOP reduction, and consequent large IOP reduction beyond 30% or even 40% in cases of advanced glaucoma.

In the last two decades, several novel classes of topical IOP-lowering drugs have been available, and now there are more choices in the treatment of glaucoma. A recent meta-analysis of the IOP-lowering effect of glaucoma drugs showed a maximum mean IOP reduction of 33% from baseline IOP in the case of monotherapy [3]. However, many patients require more than one medication to achieve adequate IOP reduction [4], [5].

More recently, to maximize patient medication adherence and quality of life, several fixed combinations of commonly used IOP-lowering medications have been developed [6]. Current commercially available, fixed combination drugs mostly include the topical beta-blocker 0.5% timolol combined with a prostaglandin analogue (PGA), an alpha-adrenoceptor agonist (AA) or a topical carbonic anhydrase inhibitor (CAI) [7]. More and more clinical trials are published to evaluate the efficacy of these fixed-combination options. However, the non-consistent results of these studies made it difficult to draw conclusions of the degree of reduction of IOP that can be achieved with different fixed-combination drugs. Therefore, to evaluate the IOP-lowering effect of the commonly used fixed-combination drugs containing timolol, a systematic review and meta-analysis was conducted, involving all relevant published randomized clinical trials in the treatment of POAG and ocular hypertension (OHT).

## Methods

### Outcome Measures

The outcome measures of efficacy were the absolute and relative IOP reductions from baseline. The standard time point of measurement was 1 month or the closest time point, with minimally 1 month and maximally 3 months. The mean diurnal IOP curve, the highest IOP decrease on the diurnal IOP curve, and the lowest IOP decrease on the diurnal IOP curve were noted [8].

### Search Strategy and Trials Selection

Randomized clinical trials were identified through a systematic search of PubMed, Embase, and the Cochrane Controlled Trials Register. The keywords for the medication were *timolol*, *dorzolamide*, *brinzolamide*, *brimonidine*, *latanoprost*, *travoprost*, and *bimatoprost*. The keywords for the disease were *glaucoma*, and *ocular hypertension*. The limit for the search was *randomized controlled trial*. The computerized searches covered the period between January 1, 1998, and September 1, 2011. Additional studies were also identified by a hand search of all the references of retrieved articles. The internet was searched using the Google^TM^ and Yahoo!^®^ search engines to obtain information.

Published clinical trials were selected based on the protocol-determined selection criteria. (i) Study design: randomized clinical trials, including parallel or crossover design. (ii) Population: over 85% of the patients had to be diagnosed with POAG or OHT. (iii) Intervention: after a medicine-free washout period, at least one of the following fixed-combination drugs, including 2% dorzolamide/0.5% timolol twice daily, 1% brinzolamide/0.5% timolol twice daily, 0.2% brimonidine/0.5% timolol twice daily, 0.005% latanoprost/0.5% timolol once daily, 0.004% travoprost/0.5% timolol once daily, and 0.03% bimatoprost/0.5% timolol once daily. (iv) Outcome variables: absolute and relative reductions from baseline in IOP. (v) Duration: at least one of time point between 1 month and 3 months.

Two reviewers (JWC, SWC) determined the trial eligibility independently. Firstly, the titles and abstracts of the obtained publications were screened. Then, full articles of the remaining identified publications were scrutinized. Only trials meeting selection criteria were assessed for methodological quality.

### Data Extraction and Qualitative Assessment

Data extraction was performed according to the customized protocol by two reviewers (JWC, SWC) independently. Any disagreement was resolved by discussion. A customized form for data extraction was used as follows. (i) Publications: the first author and published year. (ii) Method: duration, randomization technique, allocation concealment method, group design (parallel, crossover), masking (participants, investigators, examiners), country, and setting. (iii) Participants: inclusion criteria, exclusion criteria, sampling, disease types, age, sex, and withdrawals/losses to follow up (reason). (iv) Interventions: interventions (drugs, dose, route, duration), and co-medications (drugs, dose, route, duration). (v) Outcomes: definitions, measuring method, measuring time, time points, results. (vi) Statistics: simple size determination, intention-to-treat analysis, and per-protocol analysis.

Eligible studies that met inclusion criteria were rated for methodological quality by two authors independently, using a guide developed from the Delphi list for quality assessment of randomized clinical trials [3]. Each item in this quality list had the same weight. For each publication, a quality score was calculated, where “yes” was scored as 1 point for a certain quality item and “no” and “do not know” were scored as 0 point. The quality of sample studies scored out of a maximum of 18 (**Table** **1**).

**Table 1:** Quality items of the quality assessment system of methodological characteristics.*

Item code	Quality item	No. of trials scored “Yes”
A	Was a method of randomization used?	41
B	Was the treatment allocation concealed?	22
C	Were the participants blinded?	22
D	Were the investigators blinded?	33
E	Were the examiners blinded?	39
F	Were inclusion criteria specified?	41
G	Were exclusion criteria specified?	41
H	Were the interventions described explicitly?	41
I	Was comedication avoided or standardized?	41
J	Were point estimates and measures of variability presented for the primary outcome measures?	41
K	Was the period of outcome measurements equal for all groups?	41
L	Were times of IOP measurements equal for all-groups?	41
M	Was information about the method of IOP measurement presented?	41
N	Were the groups similar at baseline regarding the most important prognostic indicators?	41
O	Was it unlikely that compliance may explain differences between groups?	41
P	Was withdrawal rate reported	39
Q	Was calculation of sample size reported	33
R	Was an intention-to-treat analysis performed?	27

IOP =  intraocular pressure.

* The system was developed from the Delphi list, and was supplemented with additional items which were important for interpreting IOP measurements.

### Statistical Analysis

All statistical analyses were performed using Comprehensive Meta-Analysis software version 2.0 (Biostat, Englewood Cliffs, New Jersey) (http://www.meta-analysis.com). Outcome measure was assessed on an intention-to-treat (ITT) basis. For each study, absolute and relative IOP reductions and 95% confidence intervals (CIs) of the fixed-combination drugs were calculated. We first obtained the pooled estimates of IOP reductions with 95% CIs by fixed-combination medication using the random-effects model. Then, a mixed-effects meta-regression model was used to estimate the weighted mean differences (WMDs) in relative IOP reductions by different fixed combinations. Egger's weighted regression method was used to statistically assess publication bias.

## Results

### Eligiblity and Quality

**Figure 1:**
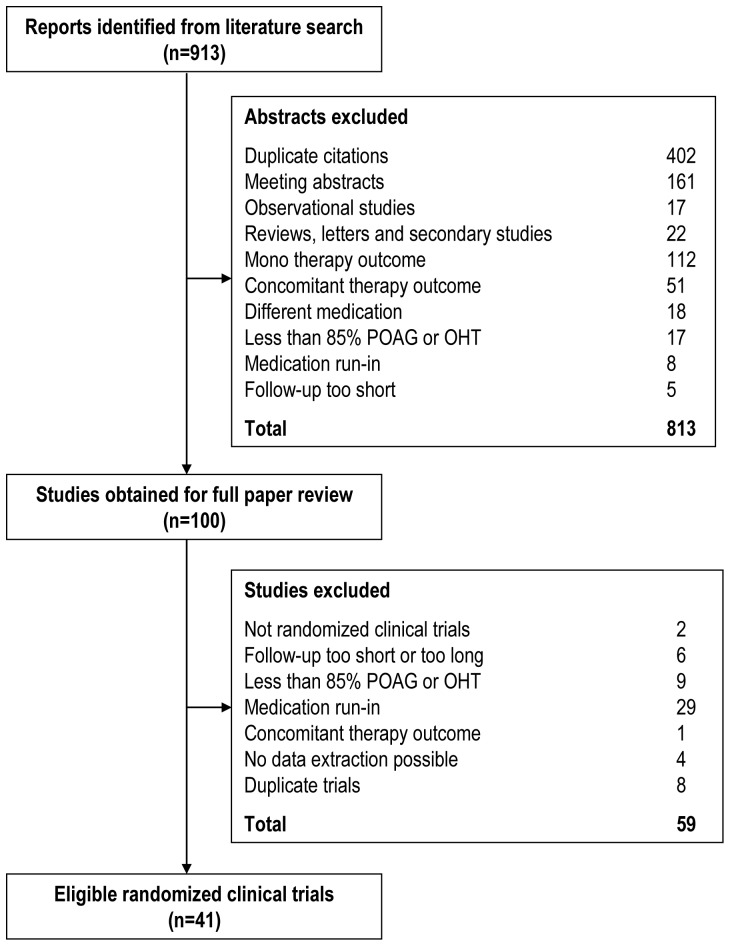
The selection flowchart of the studies included in the present meta-analysis.

The literature search identified 913 papers. Based on the content of the abstracts, 813 articles were found obviously ineligible for inclusion. From the remaining 100 articles that were retrieved for full papers, 59 had to be excluded for reasons outlined in **Figure** **1**. Finally, 41 eligible randomized clinical trials which met our inclusion criteria were included in this systematic review [9–49].

This 41 articles reported on 53 arms with six fixed combinations after medicine-free washout: 22 arms for 2% dorzolamide/0.5% timolol, 2 arms for 1% brinzolamide/0.5% timolol, 5 arms for 0.2% brimonidine/0.5% timolol, 14 arms for 0.005% latanoprost/0.5% timolol, 8 arms for 0.004% travoprost/0.5% timolol, and 2 arms for 0.03% bimatoprost/0.5% timolol.

The mean total quality score for all studies was 16.2 with a range from 13 to 18 (**Table** **2**). Twelve studies were scored less than 16, and 29 trials were scored 16 and more. There were only seven items sometimes scored as 0 point (**Table** **1**), including allocation concealment, blinding, intention-to-treat analysis, withdrawals, and sample size.

**Table 2:** Baseline characteristics of eligible randomized clinical trials.

Trial	Design	Location	Centre	Intervention			Total no.	Withdrawals (%)	Mean age (years)	Sex (M/F)	POAG or OHT (%)	Baseline IOP* (mm Hg) [mean (SD) ]	Quality score
				Medication	Route	Duration							
199810/01 [9]	DB, PG	U.S.	27	2.0% Dorzolamide/0.5% timolol	8:30 AM, 8:30 PM	3 Months	114	0.3	62.4	54/60	100	27.8 (5.0)	18
200303/01 [10]	DB, PG	U.S.	8	2.0% Dorzolamide/0.5% timolol	8 AM, 8 PM	3 Months	29	0.0	62.7	9/20	97	25.4 (3.7)	14
200304/01 [11]	DB, CR	Italy	1	2.0% Dorzolamide/0.5% timolol	8 AM, 8 PM	1 Month	20	0.0	63.0	9/11	100	24.9 (0.9)	17
200307/01 [12]	SB, CR	Greece	2	2.0% Dorzolamide/0.5% timolol	8 AM, 8 PM	6 Weeks	33	2.9	64.8	15/18	100	25.8 (1.4)	14
200402/01 [13]	DB, PG	U.S., Europe, Israel	44	2.0% Dorzolamide/0.5% timolol	8 AM, 10 PM	3 Months	273	8.8	62.8	125/148	99	-	18
200402/02 [14]	SB, PG	U.S.	30	0.005% Latanoprost/0.5% timolol 2.0% Dorzolamide/0.5% timolol	8 AM 8 AM, 8 PM	3 Months	251	4.3	63.5	112/139	98	28.4 (3.7)	16
200405/01 [15]	OL, PG	Latin America	13	2.0% Dorzolamide/0.5% timolol	8 AM, 8 PM	8 Weeks	117	6.0	61.1	47/70	92	25.0 (3.6)	15
200407/01 [16]	SB, PG	Europe	34	0.005% Latanoprost/0.5% timolol	8 AM	6 Months	163	6.0	65.5	73/90	96	27.4 (2.6)	17
200409/01 [17]	SB, PG	Spain	1	0.005% Latanoprost/0.5% timolol	9 AM	3 Months	22	0.0	63.5	9/13	100	24.7 (2.5)	13
200410/01 [18]	DB, CR	U.S.	3	0.005% Latanoprost/0.5% timolol	8 AM	6 Weeks	35	8.6	64.5	12/23	97	26.9 (3.2)	16
200505/01 [19]	DB, CR	U.S.	3	2.0% Dorzolamide/0.5% timolol	8 AM, 8 PM	8 Weeks	32	8.6	61.5	9/23	100	25.9 (2.4)	16
200507/01 [20]	DB, PG	U.S.	33	0.004% Travoprost/0.5% timolol	8 AM	3 Months	82	3.9	63.0	37/45	100	30.2 (2.7)	18
200507/02 [21]	SB, CR	Greece	1	0.005% Latanoprost/0.5% timolol	8 PM	8 Weeks	37	0.0	65.8	14/23	100	26.5 (2.8)	17
200508/01 [22]	DB, PG	U.S.	27	0.004% Travoprost/0.5% timolol	8 AM	3 Months	155	7.7	62.0	63/92	97	25.6 (2.7)	18
200510/01 [23]	DB, PG	U.S.	19	0.004% Travoprost/0.5% timolol	8 AM	3 Months	151	7.5	64.2	57/94	97	25.3 (2.3)	18
200601/01 [24]	DB, PG	Europe, Canada	53	0.005% Latanoprost/0.5% timolol	8 PM	12 Weeks	255	11.0	65.0	129/126	91	26.0 (2.3)	18
200603/01 [25]	DB, PG	France	Multi	0.004% Travoprost/0.5% timolol 0.004% Travoprost/0.5% timolol	9 AM 9 PM	6 Weeks	91	8.8	63.9	46/45	90	26.7 (3.3)	18
200603/02 [26]	SB, PG	Brazil	1	0.005% Latanoprost/0.5% timolol	7 AM	1 Month	14	0.0	59.2	-	100	22.0 (3.2)	13
200609/01 [27]	DB, PG	U.S.	53	0.2% Brimonidine/0.5% timolol	8 AM, 8 PM	12 Months	385	25.7	62.0	181/204	100	24.7 (2.7)	18
200609/02 [28]	SB, PG	Brazil	1	2.0% Dorzolamide/0.5% timolol	9 AM, 9 PM	6 Weeks	27	0.0	57.5	8/19	100	23.1 (2.1)	14
200611/01 [29]	SB, CR	Greece	1	0.005% Latanoprost/0.5% timolol	8 PM	8 Weeks	34	2.9	62.4	13/21	100	27.2 (2.9)	15
200702/01 [30]	SB, CR	Turkey	1	2.0% Dorzolamide/0.5% timolol	8 AM, 8 PM	6 Months	29	3.3	64.9	15/14	100	24.0 (2.2)	13
200704/01 [31]	SB, CR	Brazil	1	0.2% Brimonidine/0.5% timolol 2.0% Dorzolamide/0.5% timolol	8 AM, 8 PM	4 Weeks	30	0.0	56.1	12/18	100	22.9 (1.6)	16
200801/01 [32]	SB, CR	Greece	2	2.0% Dorzolamide/0.5% timolol	8 AM, 8 PM	6 Months	53	8.6	61.2	21/32	100	27.1 (2.6)	15
200804/01 [33]	DB, PG	U.S., Canada	59	0.03% Bimatoprost/0.5% timolol	8 AM	3 Months	533	6.6	62.1	247/286	100	25.9 (3.1)	18
200808/01 [34]	DB, CR	U.S.	2	2.0% Dorzolamide/0.5% timolol	8 AM, 8 PM	8 Weeks	29	3.3	68.0	11/18	100	25.1 (2.0)	16
200808/02 [35]	DB, PG	U.S.	19	2.0% Dorzolamide/0.5% timolol	8 AM, 8 PM	8 Weeks	117	6.0	61.8	47/70	99	26.2 (3.4)	18
200810/01 [36]	SB, CR	Greece	1	2.0% Dorzolamide/0.5% timolol	8 AM, 8 PM	6 Weeks	27	0.0	61.0	11/16	100	26.4 (1.6)	16
200810/02 [37]	DB, PG	U.S.	35	1.0% Brinzolamide/0.5% timolol	8 AM, 8 PM	6 Months	171	7.5	-	80/91	95	27.2 (2.7)	16
200811/01 [38]	SB, CR	Greece	2	0.2% Brimonidine/0.5% timolol	8 AM, 8 PM	3 Months	28	12.5	63.6	18/10	100	26.9 (2.8)	16
200812/01 [39]	OL, PG	Brazil, Argentina	5	0.2% Brimonidine/0.5% timolol 2.0% Dorzolamide/0.5% timolol	8 AM, 8 PM	8 Weeks	210	7.6	60.4	87/123	100	24.0 (4.0)	14
200904/01 [40]	DB, CR	Greece	1	0.004% Travoprost/0.5% timolol	10 PM	8 Weeks	34	5.9	63.9	15/19	100	28.9 (3.3)	17
200904/02 [41]	DB, PG	U.S., Europe, Australia, Singapore, Taiwan	Multi	1.0% Brinzolamide/0.5% timolol 2.0% Dorzolamide/0.5% timolol	8 AM, 8 PM	12 Months	437	10.1	64.8	181/256	91	27.3 (6.9)	18
200905/01 [42]	DB, CR	Greece	1	0.005% Latanoprost/0.5% timolol	8 PM	8 Weeks	29	3.3	63.7	13/16	100	27.7 (1.9)	16
200907/01 [43]	SB, PG	U.S.	10	0.2% Brimonidine/0.5% timolol 2.0% Dorzolamide/0.5% timolol	8 AM, 8 PM	3 Months	180	10.6	67.7	80/100	100	23.3 (4.6)	16
200910/01 [44]	OL, PG	Brazil	1	0.005% Latanoprost/0.5% timolol	8 PM	8 Weeks	18	0.0	57.8	8/10	100	24.7 (1.3)	14
200911/01 [45]	DB, PG	Europe, Turkey	31	0.004% Travoprost/0.5% timolol 2.0% Dorzolamide/0.5% timolol	9 AM 9 AM, 9 PM	6 Weeks	319	2.8	61.7	122/197	92	27.0 (3.4)	17
201002/01 [46]	DB, PG	U.S.	Multi	0.005% Latanoprost/0.5% timolol	8 PM	12 Weeks	129	11.6	64.8	57/72	98	29.0 (3.0)	18
201007/01 [47]	SB, PG	Europe	25	0.005% Latanoprost/0.5% timolol 2.0% Dorzolamide/0.5% timolol	8 PM 8 AM, 8 PM	12 Weeks	270	4.8	66.2	121/149	91	27.3 (3.7)	17
201007/02 [48]	DB, PG	U.S., Canada	45	0.005% Latanoprost/0.5% timolol	8 PM	12 Weeks	170	11.8	65.3	76/94	96	28.7 (2.6)	18
201102/01 [49]	SB, PG	Spain	1	0.005% Latanoprost/0.5% timolol 0.004% Travoprost/0.5% timolol 0.03% Bimatoprost/0.5% timolol	9 PM	12 Months	128	9.2	68.0	41/87	100	27.3 (4.0)	15

M =  male; F =  female; IOP =  intraocular pressure; SD =  standard deviation.

DB =  double blind; SB =  single blind; OL =  open label; PG =  parallel group; CR =  crossover.

* Pooled values, measurements closest to 8 AM.

The *P* values of Egger's measure of publication bias were 0.25 for mean diurnal IOP reduction, 0.13 for the highest IOP reduction, and 0.51 for the lowest IOP reduction. Because no relevant differences were observed by statistics, no publication bias was found.

### Design and Characteristics

The study design and baseline characteristics of the eligible studies are summarized in **Table** **2**. Randomized clinical trials were undertaken in Europe, U.S., Canada, Latin America, Australia, Israel, Turkey, Singapore, and Taiwan. Twenty-seven trials had a prospective, parallel design, and fourteen had a prospective, crossover design. The proportion of withdrawals varied from 0.0% to 25.7%.

Overall, 5261 patients were involved, with the mean age was 63.5 years (range from 56.1 to 68.0 years). The proportion of patients with POAG or OHT per study varied from 91% to 100%. The mean baseline IOP ranged from 22.0 mmHg to 30.2 mmHg after a medicine-free washout period.

### Intraocular Pressure Lowering Effects

Forty-four arms were reporting the mean diurnal IOP reduction; 46 arms were reporting the highest IOP reduction; and 38 arms were reporting the lowest IOP reduction. **Table** **3** gives an overview of the absolute and relative values of mean diurnal IOP reduction, and the highest and lowest IOP decrease on the diurnal IOP curve.

**Table 3:** Absolute and relative reductions in IOP for mean diurnal curve, the highest and lowest IOP decrease per study arm.

Trial	Medication	End point of measurement (weeks)	Type of measurement	Time points (hours after dosing)	Highest (SE)		Lowest (SE)		Diurnal (SE)	
					Absolute (mm Hg)	Relative (%)	Absolute (mm Hg)	Relative (%)	Absolute (mm Hg)	Relative (%)
199810/01 [9]	2.0% Dorzolamide/0.5% timolol	4	IOP Curve (2)	0, 2	9.30 (0.41)	33.70 (1.23)	8.00 (0.42)	28.20 (1.22)	8.65 (0.42)	30.95 (1.23)
200303/01 [10]	2.0% Dorzolamide/0.5% timolol	4	Single (1)	2	6.70 (0.82)	26.17 (3.22)	-	-	-	-
	2.0% Dorzolamide/0.5% timolol	6	Single (1)	2	6.30 (0.54)	25.10 (2.16)	-	-	-	-
200304/01 [11]	2.0% Dorzolamide/0.5% timolol	4	IOP Curve (8)	0, 3, 6, 9	9.50 (0.18)	38.17 (0.74)	3.90 (0.25)	18.19 (1.18)	6.10 (0.49)	26.99 (2.17)
200307/01 [12]	2.0% Dorzolamide/0.5% timolol	6	IOP Curve (6)	2, 6, 10	-	-	-	-	10.50 (0.31)	40.70 (1.20)
200402/01 [13]	2.0% Dorzolamide/0.5% timolol	4	IOP Curve (4)	0, 2, 6, 8	-	-	-	-	6.80 (0.36)	-
	2.0% Dorzolamide/0.5% timolol	4	IOP Curve (4)	0, 2, 6, 8	-	-	-	-	7.49 (0.30)	-
200402/02 [14]	0.005% Latanoprost/0.5% timolol	13	IOP Curve (3)	0, 4, 8	9.60 (0.39)	33.33 (1.37)	9.10 (0.32)	32.97 (1.17)	9.40 (0.28)	33.69 (0.99)
	2.0% Dorzolamide/0.5% timolol	13	IOP Curve (3)	0, 4, 8	8.90 (0.39)	32.36 (1.41)	8.10 (0.39)	29.03 (1.39)	8.40 (0.33)	30.55 (1.19)
200405/01 [15]	2.0% Dorzolamide/0.5% timolol	8	IOP Curve (4)	0, 2, 6, 9	7.40 (0.32)	29.60 (1.29)	5.40 (0.39)	23.89 (1.72)	6.40 (0.30)	27.12 (1.25)
200407/01 [16]	0.005% Latanoprost/0.5% timolol	4	IOP Curve (3)	0, 4, 8	-	-	-	-	9.11 (0.18)	34.51 (0.67)
200409/01 [17]	0.005% Latanoprost/0.5% timolol	4	IOP Curve (3)	0, 3, 7	6.40 (0.59)	26.12 (2.40)	6.20(0.46)	25.41 (1.88)	6.29 (0.43)	25.59 (1.75)
200410/01 [18]	0.005% Latanoprost/0.5% timolol	6	IOP Curve (3)	0, 4, 8	8.30(0.68)	30.86 (2.51)	8.00 (0.65)	30.88 (2.53)	8.20(0.51)	31.54 (1.97)
200505/01 [19]	2.0% Dorzolamide/0.5% timolol	8	IOP Curve (3)	0, 2, 8	7.30(0.64)	29.44 (2.58)	6.10 (0.57)	25.42 (2.36)	6.80(0.46)	27.31 (1.85)
200507/01 [20]	0.004% Travoprost/0.5% timolol	6	IOP Curve (3)	0, 2, 8	11.30 (0.46)	37.42 (1.52)	9.20 (0.42)	33.82 (1.54)	10.43 (0.44)	36.38 (1.53)
200507/02 [21]	0.005% Latanoprost/0.5% timolol	8	IOP Curve (6)	3, 6, 10, 14, 18, 22	10.10 (0.39)	38.11 (1.47)	6.10 (0.41)	26.87 (1.81)	7.50 (0.28)	30.99 (1.16)
200508/01 [22]	0.004% Travoprost/0.5% timolol	6	IOP Curve (3)	0, 2, 8	8.60 (0.22)	33.20 (0.86)	6.90 (0.26)	29.20 (1.13)	7.63 (0.24)	30.97 (0.99)
200510/01 [23]	0.004% Travoprost/0.5% timolol	6	IOP Curve (3)	0, 2, 8	9.40 (0.22)	37.15 (0.85)	7.40 (0.21)	32.17 (0.92)	8.37 (0.22)	34.63 (0.88)
200601/01 [24]	0.005% Latanoprost/0.5% timolol	12	IOP Curve (3)	12, 16, 20	9.10 (0.20)	35.00 (0.77)	8.20 (0.20)	33.20 (0.81)	8.70 (0.20)	34.25 (0.79)
200603/01 [25]	0.004% Travoprost/0.5% timolol	6	IOP Curve (3)	0, 2, 7	10.10 (0.68)	37.80 (2.74)	8.34 (0.63)	33.90 (2.36)	9.26 (0.68)	35.90 (2.63)
	0.004% Travoprost/0.5% timolol	6	IOP Curve (3)	12, 14, 19	9.60 (0.68)	36.10 (2.73)	8.70 (0.63)	34.40 (2.37)	9.20 (0.68)	35.20 (2.63)
200603/02 [26]	0.005% Latanoprost/0.5% timolol	4	Single (1)	3	-	-	8.50 (0.94)	38.60 (2.33)	-	-
200609/01 [27]	0.2% Brimonidine/0.5% timolol	6	IOP Curve (4)	0, 2, 7, 9	7.49 (0.17)	32.15 (0.73)	4.98 (0.20)	22.84 (0.92)	6.17 (0.19)	26.85 (0.83)
200609/02 [28]	2.0% Dorzolamide/0.5% timolol	6	IOP Curve (4)	3, 7, 11	6.20 (0.37)	26.84 (1.58)	2.90 (0.52)	15.59 (2.79)	4.50 (0.87)	21.7 (3.19)
200611/01 [29]	0.005% Latanoprost/0.5% timolol	8	IOP Curve (6)	2, 6, 10, 14, 18, 22	10.70 (0.45)	39.34 (1.66)	7.00 (0.49)	30.30 (2.11)	8.60 (0.34)	34.40 (1.36)
200702/01 [30]	2.0% Dorzolamide/0.5% timolol	4	Single (1)	4	6.50 (0.38)	27.08 (1.60)	-	-	-	-
200704/01 [31]	0.2% Brimonidine/0.5% timolol	4	IOP Curve (3)	0, 4, 8	8.20 (0.35)	34.70 (1.46)	7.50 (0.38)	33.90 (1.79)	7.80 (0.35)	34.30 (1.55)
	2.0% Dorzolamide/0.5% timolol	4	IOP Curve (3)	0, 4, 8	7.80 (0.33)	33.20 (1.43)	7.20 (0.37)	32.80 (1.66)	7.40 (0.33)	32.90 (1.57)
200801/01 [32]	2.0% Dorzolamide/0.5% timolol	8	IOP Curve (6)	2, 6, 10	9.50 (0.35)	35.06 (1.30)	5.40 (0.37)	23.18 (1.57)	7.20 (0.29)	28.57 (1.14)
200804/01 [33]	0.03% Bimatoprost/0.5% timolol	6	IOP Curve (3)	0, 2, 8	9.60 (0.16)	37.07 (0.62)	7.70 (0.17)	33.05 (0.73)	8.40 (0.14)	34.29 (0.57)
200808/01 [34]	2.0% Dorzolamide/0.5% timolol	8	IOP Curve (7)	0, 2, 4, 6, 8, 10, 12	5.90 (0.56)	24.48 (2.31)	5.30 (0.56)	21.12 (2.23)	5.80 (0.48)	23.58 (1.95)
200808/02 [35]	2.0% Dorzolamide/0.5% timolol	6	IOP Curve (8)	0, 2, 6, 10, 12	6.60 (0.30)	25.68 (1.17)	3.20 (0.30)	14.68 (1.38)	5.10 (0.30)	20.86 (1.23)
200810/01 [36]	2.0% Dorzolamide/0.5% timolol	6	IOP Curve (12)	0, 2, 4, 6, 8, 10, 12	10.10 (0.35)	37.69 (1.32)	5.40 (0.35)	25.47 (1.66)	7.30 (0.49)	32.16 (2.16)
200810/02 [37]	1.0% Brinzolamide/0.5% timolol	13	IOP Curve (5)	0, 2, 4, 8, 12	8.70 (0.29)	33.72 (1.12)	8.30 (0.30)	30.63 (1.11)	7.56 (0.33)	30.51 (1.33)
200811/01 [38]	0.2% Brimonidine/0.5% timolol	13	IOP Curve (6)	2, 6, 10	6.90 (0.43)	25.65 (1.62)	4.50 (0.36)	19.65 (1.57)	5.30 (0.23)	21.54 (0.92)
200812/01 [39]	0.2% Brimonidine/0.5% timolol	8	IOP Curve (4)	0, 2, 6, 8	7.89 (0.40)	32.93 (1.67)	6.56 (0.37)	28.52 (1.62)	7.02 (0.29)	29.96 (1.24)
	2.0% Dorzolamide/0.5% timolol	8	IOP Curve (4)	0, 2, 6, 8	7.47 (0.44)	30.96 (1.82)	6.56 (0.43)	28.64 (1.85)	6.91 (0.37)	29.49 (1.58)
200904/01 [40]	0.004% Travoprost/0.5% timolol	8	IOP Curve (6)	4, 8, 12, 16, 20, 24	11.30 (0.48)	39.10 (1.66)	7.30 (0.48)	30.04 (1.98)	9.40 (0.38)	35.34 (1.43)
200904/02 [41]	1.0% Brinzolamide/0.5% timolol	13	IOP Curve (3)	0, 2, 8	9.10 (0.35)	34.90 (1.35)	9.10 (0.36)	33.30 (1.32)	9.00 (0.35)	34.62 (1.35)
	2.0% Dorzolamide/0.5% timolol	13	IOP Curve (3)	0, 2, 8	8.80 (0.35)	33.50 (1.34)	8.70 (0.36)	31.60 (1.32)	8.75 (0.35)	33.56 (1.34)
200905/01 [42]	0.005% Latanoprost/0.5% timolol	8	IOP Curve (6)	2, 6, 10, 14, 18, 22	10.20 (0.51)	35.05 (1.76)	7.40 (0.56)	29.84 (2.26)	9.00 (0.47)	34.22 (1.80)
200907/01 [43]	0.2% Brimonidine/0.5% timolol	4	Single (1)	2	7.30 (0.56)	31.74 (2.43)	-	-	-	-
	2.0% Dorzolamide/0.5% timolol	4	Single (1)	2	7.40 (0.63)	31.36 (2.67)	-	-	-	-
200910/01 [44]	0.005% Latanoprost/0.5% timolol	8	IOP Curve (3)	12, 14, 16	10.38 (1.00)	42.09 (4.06)	8.72 (1.18)	38.76 (5.24)	9.35 (1.04)	40.34 (4.49)
200911/01 [45]	0.004% Travoprost/0.5% timolol	6	IOP Curve (2)	0, 7	10.40 (0.25)	38.66 (0.93)	8.90 (0.25)	35.46 (1.00)	9.65 (0.25)	37.06 (0.97)
	2.0% Dorzolamide/0.5% timolol	6	IOP Curve (2)	0, 7	9.30 (0.25)	34.44 (0.93)	8.50 (0.23)	33.86 (0.92)	8.90 (0.24)	34.15 (0.93)
201002/01 [46]	0.005% Latanoprost/0.5% timolol	6	IOP Curve (3)	12, 14, 20	-	-	-	-	10.10 (0.26)	36.07 (0.92)
201007/01 [47]	0.005% Latanoprost/0.5% timolol	12	IOP Curve (3)	12, 16, 20	9.80 (0.20)	36.57 (0.75)	9.60 (0.20)	36.50 (0.76)	9.70 (0.20)	36.47 (0.75)
	2.0% Dorzolamide/0.5% timolol	12	IOP Curve (3)	0, 4, 8	9.70 (0.30)	35.27 (1.09)	9.40 (0.30)	35.21 (1.12)	9.50 (0.20)	34.80 (0.73)
201007/02 [48]	0.005% Latanoprost/0.5% timolol	6	IOP Curve (3)	12, 14, 20	-	-	-	-	10.00 (0.21)	35.59 (0.75)
201102/01 [49]	0.005% Latanoprost/0.5% timolol	4	Single (1)	12	7.73 (0.70)	28.01 (2.55)	-	-	-	-
	0.004% Travoprost/0.5% timolol	4	Single (1)	12	6.56 (0.48)	24.85 (1.81)	-	-	-	-
	0.03% Bimatoprost/0.5% timolol	4	Single (1)	12	8.88 (0.58)	31.71 (2.09)	-	-	-	-

IOP =  intraocular pressure; SE =  standard error.

The pooled absolute reductions in mean diurnal IOP curve were 7.41 mmHg (95% CI, 6.69 to 8.12) for dorzolamide/timolol, 8.33 mmHg (6.82 to 9.84) for brinzolamide/timolol, 6.55 mmHg (5.59 to 7.40) for brimonidine/timolol, 8.85 mmHg (8.30 to 9.40) for latanoprost/timolol, 9.09 mmHg (8.32 to 9.87) for travoprost/timolol, and 8.40 mmHg (8.13 to 8.67) for bimatoprost/timolol (**Table** **4**). The relative mean diurnal IOP reductions were 34.9% for travoprost/timolol, 34.3% for bimatoprost/timolol, 33.9% for latanoprost/timolol, 32.7% for brinzolamide/timolol, 29.9% for dorzolamide/timolol, and 28.1% for brimonidine/timolol. Both latanoprost/timolol and travoprost/timolol were found to produce greater IOP-lowering effects than dorzolamide/timolol and brimonidine/timolol (**Table** **5**).

**Table 4:** Absolute and relative reductions in intraocular pressure.

	Time point	Absolute reduction (mm Hg)		Relative reduction (%)		No. of studies
Group		Mean	95% confidence interval	Mean	95% confidence interval	
Dorzolamide/timolol	Diurnal	7.41	6.69 to 8.12	29.9	27.4 to 32.4	18
	Highest	8.03	7.36 to 8.71	31.3	29.3 to 33.3	19
	Lowest	6.31	5.15 to 7.46	25.9	22.4 to 29.4	15
Brinzolamide/timolol	Diurnal	8.33	6.82 to 9.84	32.7	28.3 to 37.1	2
	Highest	8.86	8.43 to 9.30	34.2	32.5 to 35.9	2
	Lowest	8.68	7.89 to 9.46	31.9	29.3 to 34.5	2
Brimonidine/timolol	Diurnal	6.55	5.59 to 7.40	28.1	23.2 to 32.9	4
	Highest	7.59	7.19 to 7.99	31.5	28.7 to 34.3	5
	Lowest	5.87	4.58 to 7.16	26.1	20.6 to 31.6	4
Latanoprost/timolol	Diurnal	8.85	8.30 to 9.40	33.9	32.5 to 35.2	12
	Highest	9.29	8.67 to 9.91	34.5	32.5 to 36.6	10
	Lowest	7.86	7.02 to 8.70	32.0	29.6 to 34.5	9
Travoprost/timolol	Diurnal	9.09	8.32 to 9.87	34.9	33.0 to 36.8	7
	Highest	9.49	8.66 to 10.32	35.5	32.8 to 38.3	8
	Lowest	7.99	7.34 to 8.65	32.6	30.5 to 34.6	7
Bimatoprost/timolol	Diurnal	8.40	8.13 to 8.67	34.3	33.2 to 35.4	1
	Highest	9.46	8.89 to 10.02	34.8	29.6 to 40.0	2
	Lowest	7.70	7.36 to 8.03	33.1	31.6 to 34.5	1

**Table 5:** Weighted mean difference in relative intraocular pressure reductions.*

Time Point	Treatment comparison		Weighted mean difference (%)		*P* value
	A	B	Mean	95% confidence interval	
Diurnal	Latanoprost/timolol	Dorzolamide/timolol	3.8	0.8 to 6.7	0.011
	Latanoprost/timolol	Brimonidine/timolol	5.9	2.5 to 9.4	0.001
	Travoprost/timolol	Dorzolamide/timolol	3.3	2.2 to 4.5	0.000
	Travoprost/timolol	Brimonidine/timolol	7.0	2.5 to 11.6	0.003
Highest	Travoprost/timolol	Dorzolamide/timolol	4.2	0.6 to 7.8	0.021
	Bimatoprost/timolol	Dorzolamide/timolol	3.6	2.3 to 5.0	0.000
Lowest	Latanoprost/timolol	Dorzolamide/timolol	6.2	1.4 to 10.9	0.011
	Latanoprost/timolol	Brimonidine/timolol	6.0	0.9 to 11.1	0.021
	Travoprost/timolol	Dorzolamide/timolol	6.7	1.5 to 12.0	0.012
	Travoprost/timolol	Brimonidine/timolol	6.6	1.9 to 11.4	0.006

* For comparisons of treatment A versus treatment B, statistically significant results are shown, and a weighted mean difference above 0 indicates that relative IOP reduction is greater for treatment A than for treatment B.

The absolute values of the highest IOP reductions varied from 7.59 mmHg for brimonidine/timolol to 9.49 mmHg for travoprost/timolol, and the relative reductions ranged from 31.3% for dorzolamide/timolol to 35.5% for travoprost/timolol **(** **Table** **4** **)**. Travoprost/timolol and bimatoprost/timolol produced greater relative reductions than dorzolamide/timolol, with WMDs being 4.2 (0.6 to 7.8), 3.6 (2.3 to 5.0) respectively (Table 5).

The pooled results of absolute and relative values of the lowest IOP reductions of six fixed combinations are also shown in **Table** **4**. Travoprost/timolol was significantly more effective in lowering IOP than dorzolamide/timolol (WMD: 6.7; 95% CI, 1.5 to 12.0), and brimonidine/timolol (WMD: 6.6; 95% CI, 1.9 to 11.4); and latanoprost/timolol also was significantly more effective than dorzolamide/timolol (WMD: 6.2; 95% CI, 1.4 to 10.9) and brimonidine/timolol (WMD: 6.0; 95% CI, 0.9 to 11.1) (**Table** **5**).

## Discussion

This systematic review and meta-analysis of data from 40 randomized clinical trials reveal that all six commonly used fixed-combination drugs containing 0.5% timolol can effectively lower IOP in patients with POAG and OHT. After completely washing out all medication, the mean diurnal IOP reductions ranged from 6.55 mmHg for brimonidine/timolol to 9.09 mmHg for travoprost/timolol; the highest IOP reductions varied from 7.59 mmHg for brimonidine/timolol to 9.49 mmHg for travoprost/timolol; and the lowest IOP reductions ranged from 5.87 mmHg for brimonidine/timolol to 7.99 mmHg for travoprost/timolol.

The overview of relative IOP reductions at diurnal curve showed that travoprost/timolol, bimatoprost/timolol, and latanoprost/timolol were the three most effective fixed-combinations. The mixed-effects meta-regression results revealed that latanoprost/timolol and travoprost/timolol were more effective than dorzolamide/timolol and brimonidine/timolol. However, the difference for bimatoprost/timolol was not statistically significant, which might be a “negative” result because that the data is based on only one single trial [50]. For the highest IOP reduction, travoprost/timolol and bimatoprost/timolol were more effective than dorzolamide/timolol. Latanoprost/timolol and travoprost/timolol were also more effective than dorzolamide/timolol and brimonidine/timolol in the lowest IOP reduction. Therefore, both latanoprost/timolol and travoprost/timolol might achieve better IOP-lowering effects among the six fixed-combination agents.

The overview of relative results of mean diurnal IOP reduction, the highest and lowest IOP reduction found that brinzolamide/timolol achieved an IOP-lowering effect of more than 30%. However, the mixed-effects meta-regression results suggested that there was no significant difference in lowering IOP when comparing brinzolamide/timolol with dorzolamide/timolol and brimonidine/timolol. The pooled data of brinzolamide/timolol are based on only two papers. One trial found that 1% brinzolamide/0.5% timolol was superior in IOP-lowering efficacy to either brinzolamide 1% or timolol 0.5% [37]. The other trial suggested that the IOP-lowering efficacy of brinzolamide/timolol was noninferior to dorzolamide/timolol [41]. Owing to the “small-study effects” with the presence of substantial between-study heterogeneity, it might not be the truly IOP-lowering effect of brinzolamide/timolol.

A previous meta-analysis including 28 randomized clinical trials evaluated the IOP lowering effects of all commonly used mono-therapies in patients with POAG and OHT, and revealed that the relative peak IOP reductions were 33% for bimatoprost, 31% for latanoprost, 31% for travoprost, 27% for timolol, 25% for brimonidine, 22% for dorzolamide, and 17% for brinzolamide [3]. The present meta-analysis found that when using as fixed combinations with timolol, dorzolamide/timolol, brinzolamide/timolol and brimonidine/timolol can result an IOP-lowering effect of more than 30%. However, the relative IOP reductions of the fixed combinations of 0.5% timolol and PGAs were only 34.8% for latanoprost/timolol, 33.0% for travoprost/timolol, and 32.9% for bimatoprost/timolol. One explanation is that with any fixed combination of 0.5% timolol and a PGA, a timolol dose will be omitted, leading to a lower IOP reduction [8], [51]. Because timolol has the peak effect approximately 2 hours after dosing, and prostaglandins provide maximal IOP reduction during the last half of the dosing interval (ie, post instillation hours 12 through 24) [52], the peak effect of prostaglandin-timolol fixed combinations might be provided by prostaglandins mostly, but not the combination of prostaglandins and timolol. Another explanation is that the terminology concerning *diurnal* is not consistent in the studies reporting a mean of several IOP measurements during a (part of a) day, and only a limited number of measurements during only a part of a 24-hour period are achieved [8]. Nineteen arms from 18 trials reported a mean diurnal IOP curve of the fixed combination of timolol and a PGA. In 10 arms, all measurements were obtained within 8 hours after dosing, with three moments in 9 trials and two moments in the other one. In 6 arms, measurements were obtained in three moments up to 12 to 24 hours after installation. Full 24-hours IOP measurements were obtained in only 4 trials. If one includes only IOP measurements within a period of 8 hours or fewer after the administration of a combination of timolol and a PGA, the absence of peak efficacy moments of the PGA will lead to an underestimation of IOP-lowering effect [8].

Although we tried to conduct a thorough review of the existing literature, this present analysis has limitations inherent to any systematic review. First, a limitation of this meta-analysis is that only published studies were included. Although multiple databases and websites were searched, unfortunately, it is possible that we may have failed to include some papers, especially those published in other languages. A specific limitation of this analysis is that many trials lacked adequate allocation concealment, blinding, sample size assessment, and intention-to-treat analysis, which may leave them vulnerable to bias and misestimation of the beneficial effects of IOP-lowering agents. Finally, the pooled data of bimatoprost/timolol and brinzolamide/timolol are based on only two papers. Therefore, more research is still needed on the available guidance derived from the currently literature.

Lowering IOP is beneficial in both POAG and OHT. Depending on the glaucomatous damage and the presence of other risk factors, the target IOP often has to be chosen such that IOP lowering beyond 30% or even 40% is necessary. However, the maximum mean IOP reduction from baseline IOP was 33% in the case of monotherapy [3]. Therefore, the fixed-combination medications are needed to reach these low target IOP levels, which not only provide better IOP-lowering effects, but also improve compliance and eliminate the washout effect.

In conclusion, the results of this systematic review suggest that all six commonly used fixed-combination drugs containing timolol can effectively lower IOP in patients with POAG and OHT, and both latanoprost/timolol and travoprost/timolol might achieve better IOP-lowering effects among the six fixed-combination agents.
